# Co-Occurrence and Clustering of Sedentary Behaviors, Diet, Sugar-Sweetened Beverages, and Alcohol Intake among Adolescents and Adults: The Latin American Nutrition and Health Study (ELANS)

**DOI:** 10.3390/nu13061809

**Published:** 2021-05-26

**Authors:** Ana Carolina B. Leme, Gerson Ferrari, Regina M. Fisberg, Irina Kovalskys, Georgina Gómez, Lilia Yadira Cortes, Martha Cecilia Yépez Gárcia, Marianella Herrera-Cuenca, Attilo Rigotti, María Reyna Liria-Domínguez, Mauro Fisberg

**Affiliations:** 1 Center for Excellence in Nutrition and Feeding Difficulties, PENSI Institute, Sabará Children’s Hospital, São Paulo 05076-040, Brazil; mauro.fisberg@gmail.com; 2 Family Relations and Applied Nutrition, University of Guelph, Guelph, ON N1G 2W1, Canada; 3 Escuela de Ciências de la Actividad Fisica, el Deporte y la Salud, Universidad de Santiago de Chile (USACH), Santiago 7500618, Chile; gersonferrari08@yahoo.com.br; 4 Department of Nutrition, School of Public Health, University of São Paulo, São Paulo 05508-060, Brazil; regina.fisberg@gmail.com; 5 Faculty of Medicine, Pontifical Catholic University from Argentina, Buenos Aires C1107AAZ, Argentina; ikovalskys@gmail.com; 6 Department of Biochemistry, School of Medicine, University of Costa Rica, San José 11501-2060, Costa Rica; georgina.gomez@ucr.ac.cr; 7 Department of Nutrition and Biochemistry, Pontifical University Catholic from Javeriana, Bogota 111321, Colombia; ycortes@javeriana.edu.co; 8 College of Health Science, University of San Francisco Quito, Quito 170901, Ecuador; myepez@usfq.edu.ec; 9 Center of Developmental Studies, Central University of Venezuela (CENDES-UCV)/Bengoa Foundation, Caracas 47604, Venezuela; marianella.herrera@ucv.ve; 10 Center of Molecular Nutrition and Chronic Diseases, Department of Nutrition, Diabetes and Metabolism, School of Medicine, Pontifical Catholic University from Chile, Santiago 833115, Chile; arigotti@med.puc.cl; 11 Investigacíon Nutricional, La Molina, Lima 15024, Peru; rliria@iin.sld.pe; 12 Facultad de Ciencias de la Salud, Universidad Peruana de Ciências Aplicadas, Lima 15023, Peru

**Keywords:** health behaviors, diet, sedentary behavior, alcohol intake, sugar-sweetened beverages, cluster-analysis, ELANS study

## Abstract

Poor diet, sedentary behaviors, sugar-sweetened beverages (SSB) and alcohol intake seem to co-exist in complex ways that are not well understood. The aim of this study was to provide an understanding of the extent to which unhealthy behaviors cluster in eight Latin America countries. A secondary aim was to identify socio-demographic characteristics associated with these behaviors by country. Data from adolescents and adults from the “Latin American Health and Nutrition Study” was used and the prevalence of screen-time, occupational and transportation–sedentary time, socializing with friends, poor diet, SSB and alcohol intake, alone and in combination, were identified. The eight Latin America (LA) countries added to analyses were: Argentina, Brazil, Chile, Colombia, Costa Rica, Ecuador, Peru, and Venezuela. Logistic regression was used to estimate associations between ≥2 behaviors clustering, socio-demographics and weight status. Among 9218 individuals, the most prevalent behaviors were transportation and occupation–sedentary time, SSB and alcohol intake. Younger, female, married/living with a partner, low and middle-income and obese individuals had higher chances for these clustering behaviors. These results provide a multi-country level of understanding of the extent to which behaviors co-occur in the LA population.

## 1. Introduction

Obesity and non-communicable diseases (NCDs) are the leading causes of death worldwide [1]. Latin American (LA) countries are not impervious to this health condition, with a prevalence of around 30% for overweight and 25% for obesity. Chile has the highest prevalence for overweight (37.8%) and obesity (30.8%) [2]. Furthermore, cases of other NCDs, such as cardiovascular diseases with a prevalence of 31% and cancer with 14%, are numerous in Latin American countries [3]. While non-modifiable mechanisms (i.e., genetics) are partly to blame, there is a strong rationale for the influence of modifiable factors such as sedentary behaviors, dietary intake, and physical inactivity in the genesis of obesity [4].

High intake of energy-dense food items, including sugar sweetened beverages (SSB), ready-to-eat meals, and savory snacks [2], low intake of fruit and vegetables (FV) and other whole-grain items [5], low levels of moderate-to-vigorous physical activity (MVPA) [6], and high-time spent in sedentary behaviors [7] have individually been associated with adolescents’ and adults’ overweight status and obesity in a large number of studies. However their effects on individuals’ lifestyles presented multi-variables and were interrelated [8]. Clustering lifestyle behaviors and the association of unhealthy weight gain in children [9] and adolescents [8,9] from high-income countries (HIC) has led to some understanding of the potential interplay among different behavior patterns. However, there is a lack of studies targeting adults and those from low- and middle-income countries (LMIC), including the LA countries.

Healthy and unhealthy behaviors (i.e., energy-related behaviors (EBRB)) seem to co-exist in complex ways that are not well understood [8]. Although previous evidence is informative, there are some limitations that should be taken into account. Most of the studies look solely at the analysis of single lifestyle behaviors or at a few artificial combinations of lifestyle behaviors, and thus can hinder current understanding as to how these lifestyle risk factors naturally group together to predict early adverse outcomes. Cluster analysis is a multivariate data-analysis method for organizing individuals into homogenous groups according to their similarities in a number of measured characteristics and behaviors. It is feasible to identify which lifestyle behaviors coexist among individuals, permitting a more integrated approach to comprehending possible behavioral risk-factor combinations that may then be directed toward promoting the health of individuals. Evidence of clustering health behaviors has recently gained attention [8,10,11,12]. Nevertheless, how EBRB, including diet and sedentary behaviors, naturally cluster together and contribute to adverse health effects is currently unknown in adolescents and adults from LMIC.

Previous evidence that focuses on a single country for data analysis do not allow extrapolation of findings beyond the setting where the study was conducted, and the use of different designs and methodologies make comparison between studies difficult [13]. Multi-country studies to standardized methods across countries allows international generalizability in order to better understand associations for further development of public health policies and behavioral-change strategies [14]. Furthermore, differences between HIC and LMIC may be revealed in terms of sedentary behaviors and dietary intake. Adolescents and adults may be accessing their electronic devices while eating, and this might be impacting on their diet quality. For example, increased time spent before the TV and/or computer may be associated with poor diet quality. Usually, HICs in north America have the habit of eating while working/studying in front of electronic devices, and some LA countries still pause their work/study to take their meals. Nevertheless, the accelerated globalization process may attenuate these lifestyles in LA [12].

Thus, the aim of this study was to provide a population level understanding of the extent to which EBRB (sedentary behaviors, poor diet quality, excessive SSB, and alcohol intake) cluster in eight LA countries using a multi-country representative sample of adolescents and adults. A secondary purpose was to identify socio-demographic characteristics associated with unhealthy behaviors clustering by country. Individuals from LA countries are a highly vulnerable population [15], with changes in lifestyle and environment underlying the need for a better understanding of their sedentary behaviors and dietary patterns for the development of successful health policies and behavioral-change strategies.

## 2. Materials and Methods

### 2.1. The Latin American Health and Nutrition Survey (ELANS) Overview

The Latin American Health and Nutrition Survey (acronym in Spanish ELANS) is a household-based multi-national cross-sectional survey. ELANS aimed to identify the weight status and lifestyle behaviors of a representative population from eight LA countries: Argentina, Brazil, Chile, Colombia, Costa Rica, Ecuador, Peru, and Venezuela. This represents approximately 60% of the total countries in LA. Data collection was carried out from September 2014 to July 2015. The study protocol was approved by the Western Institutional Review Board and is registered in ClinicalTrials.gov. Each country’s protocol was also approved by their respective Institutional Review Boards (IRB). All participants provided written consent/assent forms prior to their participation in the study. Participant confidentiality for the pooled data was maintained using identification codes rather than names. All data transfer was performed with a secure file sharing system.

### 2.2. Sample

The sample consisted of 9218 individuals aged 15 to 65 years from an urban population living in LA countries. This was a random complex multistage sample, stratified by geographical region, sex, and socio-economic status (SES), with a random selection of primary and secondary sampling units. The households were selected within each secondary sampling unit and through a systematic randomization. Selection of a respondent within a household was done employing 50% of the sample next birthday [16], 50% last birthday [17] methods, controlling quotas for sex, age, and SES. The representative sample size was based on a confidence interval of 95% and maximum error of 3.49%. Sampling weighting was applied at each country level. SES was assessed using a country-dependent questionnaire taking into consideration legislative requirements or established local standards. Details of the study design and protocol can be found elsewhere [18].

### 2.3. Socio-Demographic Factors

Independent variables included socio-demographic characteristics. Individuals’ characteristics included age groups. The World Health Organization (WHO) defines adolescents as those people between 10–19 years of age [19]. Therefore, data from adolescents aged 15–19 years participating in the ELANS were included. Adults were the other age group included in the ELANS study—from 20 to 65 years old.

Other participants’ characteristics included sex (female vs. male), race/ethnicity (Caucasian vs. non-Caucasian), educational background (≤high-school and some college/university degree), and marital status (married/living with a partner versus single/not living with a partner). SES was divided into three categories: low, medium, and high-based on the national indexes used in each country [18].

### 2.4. Weight Status

Weight and height measurements were carried out by trained research assistants following a standardized protocol for anthropometric procedures and collection drawn up by the ELANS group [18]. Participants were asked to wear normal, light indoor clothing and remove their shoes and other personal belongings. Body weight was measured in kilograms, to the nearest 0.1 kg, with portable digital scales. Height was measured in centimeters with stadiometers and the reading taken to the last completed 0.1 cm. Body mass index (BMI) was calculated from the formula: weight (kg)/height^2^ (m). Adolescents’ weight status was classified according to the WHO z-scores for age and sex [20] in underweight (≥−2 z-score), normal weight (−2 to 1 z-score), overweight (1 to 2 z-score), and overweight (≥2 z-score). Adults (≥20 years old) were classified according to the WHO for this population group [21].

### 2.5. Energy–Balance Related Behaviors (EBRB)

EBRB included sedentary behaviors, diet intake, sugar sweetened beverages (SSB) and alcohol intake. SSB intake was noted in addition to diet diversity score, as this might be an independent indicator of poor health behavior. EBRB were assessed by self-reported questionnaires for adolescents and adults.

#### 2.5.1. Sedentary Behaviors

Participants reported an average time (hours and minutes) they spent in seven sedentary behaviors (SB) categories while sitting during the last 7 days: (“On average, how many minutes do you spend doing the following activities while sitting”) (i) watching TV, (ii) using the computer, (iii) using videogame, (iv) reading, (v) socializing with friends, (vi) talking on the phone, and (vii) driving. Subsequently, all primary SB were assigned to one of the following activities domains: screen-time, occupation-time, and transportation-time [22]. Screen-time was the sum of time spent on watching TV and computer use. The proxy categories for occupation and transportation times were identified for reading and driving, respectively. Socializing with friends while sitting was used as a single item variable. Leisure-time activities were identified using the sum of proxy categories: TV time, computer use, talking on the phone, and socializing with friends. All the participants exceeded the cut-off proposed. Thus, in the current study the option was to use the three domains of SB and the single item “socializing with friends”. For adults all of these domains did not have specific cut-off points. For adolescents, the domains socializing with friends, talking on the phone, and driving, also did not have specific cut-off points. There was a smaller proportion of adolescents (13.3%) as compared to adults (86.7%). In order to maintain consistency with the cut-off points, it was opted to provide the median for meeting/not meeting recommendations for specific SB domains for each population. Therefore, the cut-off points for each domain were: 150 min for screen-time, 30 min for occupation-time, 60 min for transportation-time, and 60 min for socializing with friends.

#### 2.5.2. Dietary Intake

Dietary intake data was obtained from two in-person 24 h dietary recall (24 h DR) interviews using an automated multiple-pass method [23]. The food and beverage intake recorded was converted into energy, macronutrient, and micronutrient values using the Nutrition Data System for Research software (NDS-R version 2013) [24]. The NDS-R software is based on the US Department of Agriculture food composition table, thus, a standardized procedure matching local foods to the USDA composition table was performed by trained dietitians in each country in order to minimize errors. A concordance rate between 80% and 120% for energy and macronutrients was found when comparing the USDA and each country’s food composition table [25].

Dietary intake was assessed using the “Dietary Diversity Score (DDS)”. This method provided better response in comparison to other dietary indexes, such as Dietary Quality Score [26], and was developed by the Food and Agriculture Organization (FAO) to assess household and individual dietary diversity [27]. The household DDS was meant to reflect the economic ability of a household to access a variety of foods. Evidence has shown that an increase in dietary diversity is associated with SES and household food security [28]. Alternatively, individual DDS aimed to reflect nutrient adequacy. Research into different age groups has been associated with an increased nutrient adequacy of diet and DDS. For the purpose of this study, DDS was calculated at the individual level. DDS classified foods in nine groups: (i) cereals, (ii) white roots and tubers, (iii) vegetables, (iv) fruits, (v) meat, poultry, and offal, (vi) fish and seafood, (vii) eggs, (viii) pulses, legumes, and nuts; and (ix) milk and dairy products. The consumption of 15 g for each food group (equivalent to a tablespoon) [29] was the cut-off point for meeting or not meeting the recommendation and a score based on meeting at least five groups was created: 1 meeting and 0 not meeting. Previous details on how this measurement was adapted to the Latin American context has been published elsewhere [26].

#### 2.5.3. Beverage Intake

Beverage intake was assessed using the Beverage Intake Questionnaire (BEVQ) designed to obtain the frequency of beverage intake across 10 beverage categories: water, flavored water, soft-drinks, fruit drinks, sport drinks, energy drinks, tea and coffee, other non-alcoholic drinks, and alcoholic drinks. For each beverage, participants answered whether they consume the specific category of beverage, the frequency of intake (daily, weekly, monthly), and how often they drink the selected unit (1–10 occasions). The list of beverages included in the questionnaire was standardized across the ELANS countries; however, regional variations in beverage consumption patterns required some cultural and regional adaptation for some items within the beverage categories [18]. For the purpose of this study, only the categories of sugar sweetened beverages (SSB) and alcoholic beverages were included. SSB included soft-drinks, fruit drinks, and sports and energy drinks. Alcoholic beverages were deemed as beer, wines, and liquor and cocktails [30,31]. The sum of these two groups of beverages was calculated and then the median for these two groups were calculated to provide a cut-off point for meeting the recommendations. The cut-off point for SSB was 4.5 glasses and for alcohol beverages 0.5 of a glass [32].

### 2.6. Statistical Analysis

All analyses were performed using the statistical software SAS studio (version 5.2). Descriptive statistics of the study population were calculated as mean (standard error) for continuous variables and frequency (%) for categorical variables. Proportions of all ELANS participants and by country, with unhealthy levels of each behavior, alone and in combination, were calculated to identify the most common patterns. Logistic regression was used to estimate association of EBRB clustering (≥2 unhealthy behaviors versus 0–1 unhealthy behavior) with the socio-demographic characteristics described above, stratified or not by country. Marginal standardization to transform regression coefficients into standardized proportions were used. For all analyses, 5% was considered a significant level (*p* < 0.05).

## 3. Results

### 3.1. Sample Characteristics

The final dataset consisted of 9218 adolescents and adults (52.17% female) from eight Latin America countries with complete data on socio-demographic and all of the five EBRB indicators. The ELANS sample characteristics in terms of socio-demographics, lifestyle behaviors, and weight status are displayed in Table 1, separately for each LA country. Mean age of the entire population was 35.82 ± 0.15 years. Venezuela had the highest educated sample, as nearly 19% of the participants were undergraduate or held a bachelor’s degree, while Argentina was the lowest (4.27%). Brazil had the highest number of individuals reporting being on a high-income (35.25%) while Venezuela was the most low-income population (77.74%). A high proportion of all the ELANS sample reported themselves as being non-Caucasian (63.26%), with Ecuador and Argentina reporting the highest (95.12% and 71.82%, respectively). The highest prevalence of overweight was found in Peru (38.26%) and obesity in Chile (30.83%).

The average for total energy intake consumed was 1992.93 ± 6.47 kcal/day with a higher intake in Ecuador (2212.55 ± 21.34 kcal/day) and a lower in Chile (1732.72 ± 18.49 kcal/day). For sedentary time, mean screen-time in all ELANS sample was 192.65 ± 1.95 min/day, and Costa Rica (238.11 ± 8.58 min/day) and Ecuador (140.17 ± 3.97 min/day) spent the highest and lowest time, respectively. Occupational mean time was 55.29 ± 1.09 min/day. Costa Rica spent the highest (75.95 ± 5.23 min/day) and Ecuador the lowest (38.13 ± 2.23 min/day) time in occupational sedentary time. Transportation mean time for all the ELANS sample was 87.25 ± 2.01 min/day, and Costa Rica and Chile had the highest (130.73 ± 10.53 min/day) and lowest time (63.95 ± 3.87 min/day), respectively. Average time in the ELANS for socializing with friends was 92.71 ± 1.15 min/day. Argentina (107.04 ± 3.02 min/day) spent the highest time and Ecuador (64.02 ± 2.53 min/day) the lowest.

### 3.2. Individual Energy–Balance Related Behaviors (EBRB)

**Figure 1:**
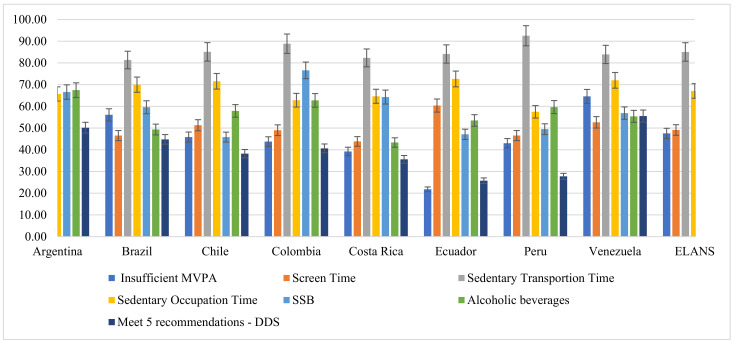
Prevalence (%) of sedentary, drinking behaviors and meeting the five recommendations for DDS in adolescents and adults from the Latin American Health and Nutrition Study (ELANS) (*n* = 9218).

The prevalence of energy–balance related behaviors in the overall ELANS population and each country are presented in Figure 1. The most prevalent individual unhealthy behavior was time spent on transportation while sitting (85.05%) and Peru (92.54%) showed the highest prevalence among the LA countries. The least prevalent individual behavior for the total ELANS was not meeting the five recommendations for the DDS with 40.92%, and for individual countries Venezuela showed the highest (55.53%) and Ecuador the lowest (25.75%) prevalence for this dietary behavior. Overall, screen-time had a prevalence of 49.12%, with Ecuador showing the highest (60.38%) and Costa Rica the lowest (43.86%) prevalence. Occupation time while sitting was reported by 67.11% in the whole ELANS sample, and Ecuador (72.63%) and Peru (57.50%) had the highest and lowest prevalence. Sugar Sweetened Beverages had a prevalence of 59.29% in the entire ELANS sample, Colombia (76.59%) and Chile (45.85%) being the most and least prevalent for SSB intake, respectively. Alcoholic beverages intake had a prevalence of 56.27%, and Argentina (67.47%) had the highest and Costa Rica (43.36%) the lowest prevalence among all the Latin American Countries.

### 3.3. EBRB Clustering

EBRB clustering, defined as having at least two unhealthy behaviors, were shown in 41.25% of all the ELANS sample. Colombia had the highest prevalence (49.27%), while Ecuador had the lowest prevalence (33.88%) (Table 2). In the entire ELANS sample, the most common unhealthy behavior pairs were found for SSB and alcohol intake (34.89%), diet and SSB intake (34.58%), and screen-time and SSB intake (30.49%). The three most common unhealthy behavior pairs in Argentina were SSB and alcohol intake (45.50%), socialization with friends + alcohol intake (39.18%), and socialization with friends + SSB intake (37.05%). In Brazil these were diet + SSB intake (33.30%), socialization with friends + screen-time (30.80%), and SSB + alcohol intake (30.15%); in Chile, diet + alcohol intake (37.54%), screen-time + diet (31.06%), and SSB + alcohol intake (29.12%); in Colombia, SSB and alcohol intake (48.29%), screen-time + SSB intake (38.05%), and socialization with friends + SSB intake (37.32%); in Costa Rica, diet + SSB intake (40.60%), screen-time + SSB intake (35.84%) and screen-time + diet (35.46%); in Ecuador, diet + alcohol intake (39.88%), diet + SSB intake (35.00%), and screen-time + diet (29.88%); in Peru, diet + alcohol (42.86%), screen-time + diet (38.54%), and diet + SSB (35.76%); and in Venezuela, SSB + alcohol intake (33.22%), diet + alcohol intake (26.33%), and socialization with friends + alcohol intake (26.41%).

### 3.4. EBRB and Socio-Demographic Characteristics

Table 3 presents results of the entire ELANS sample and country-stratified logistic regressions, in the form of predicted proportions of the LA population reporting ≥2 unhealthy behaviors across different socio-demographic groups. Among the ELANS sample, age was positively associated with clustering ≥2 unhealthy behaviors (OR 0.01; 95%CI 0.01, 0.02). Other results were as follows: being female (OR 0.59; 95%CI 0.51, 0.68), ≤high-school (OR 0.68; 95%CI 0.54, 0.82), being single or living alone (OR 0.27; 9%%CI 0.35, 0.18), being from low-income status (OR 0.66; 95%CI 0.53, 0.78) and middle-income status (OR 0.40; 95%CI 0.28, 0.53), and being normal weight (OR −0.18; 95%CI −0.30, −0.08).

In Argentina there was a positive association with age (OR 0.01; 95%CI 0.00, 0.02), being a female (0.55; 0.33, 0.78), ≤high school (0.80; 0.21, 1.39), self-identified as Caucasian (0.34; 0.09, 059), being from low-income (0.82, 0.30, 1.37) and middle-income (0.54; 0.02, 1.09), in that these individuals reported less unhealthy behaviors as compared to their counterparts.

In Brazil, associations were found for age (0.01; 0.01, 0.02), being female (0.59; 0.41, 0.77), ≤high-school (1.19; 0.86, 1.53), low-income (1.04; 0.75, 1.36) and middle-income (0.50; 0.31, 0.69) status.

Chile was associated with being female (0.56; 0.28, 0.84), ≤high-school (0.67; 0.21, 1.13), being low-income (0.97; 0.49, 1.46) and middle-income (0.57; 0.09, 1.06) and reporting unhealthy behaviors in comparison to one’s counterparts. Being underweight (−0.52; −0.88, −0.15) and normal weight (0.51; 0.88, 0.15) were also negatively associated with unhealthy behaviors (as compared to obese individuals).

In Colombia presenting ≥2 unhealthy behaviors was associated with being female (0.06; 0.28, 1.04) and being of low-income status (0.62, 0.11, 1.14). Being single/living alone was negatively associated (0.44; 0.67, 0.20).

Presenting ≥2 unhealthy behaviors in Costa Rica was positively associated with age (0.02; 0.01, 0.03), being female (0.74; 0.46, 1.03) and being of low-income status (0.96; 0.49, 1.42) and negatively associated with being single/living alone (0.42; 0.70, 0.13).

The population from Ecuador showing ≥2 unhealthy behaviors was positively associated with age (0.02; 0.01, 0.03), being female (0.88; 0.58, 1.18), ≤high-school (0.97, 0.39, 1.54), low-income (1.26; 0.68, 1.85) and middle-income (0.75; 0.33, 1.17); and negatively associated with being single/living alone (0.47; 0.77, 0.18) and being normal weight (0.39; 0.78, 0.01).

Peru was positively associated with age (0.01; 0.01, 0.02), being female (0.66, 0.42, 0.90), ≤high-school (0.42, 0.02, 0.82), being low-income (0.95, 0.63, 1.26) and middle-income (0.73; 0.38, 1.07) status; and negatively associated with being single/living alone (0.42, 0.69, 0.21) and being normal weight (0.49, 0.81, 0.16).

In Venezuela, presenting ≥2 unhealthy behaviors were associated with age (0.01; 0.00, 0.02), being female (0.42; 0.18, 0.67), ≤high-school (0.66; 0.36, 0.97), and being of low-income status (0.57; 0.04, 1.08).

## 4. Discussion

Existing studies have examined clustering of EBRB in youth [8] and adults [10,11] from HIC, but have not examined how these patterns differs across LMIC in adolescents and adults. In this multi-country sample of eight LA countries, unhealthy behaviors were highly prevalent, particularly for transportation- and occupation-sedentary time, SSB intake, and alcohol intake. Furthermore, these unhealthy behaviors are often a co-occurring pattern that are more prevalent in some countries than others, with nearly half of Argentina and Colombia and nearly a third for Chile, Ecuador and Venezuela reporting ≥2 unhealthy behaviors. This EBRB clustering was more common among certain socio-demographic groups; younger and female individuals, married/living with partner, and low- and middle-income individuals. Obese participants also tend to present more chances of these clustering behaviors.

Evidence from cross-sectional and longitudinal studies [8,10,11,12,14] corroborates with this study, showing associations between poor dietary intake and sedentary time, alcohol and SSB intake. Further, there are some notable differences across countries in the clustering of these behaviors. For example, results showed that alcoholic intake clustering was more prevalent in Argentina than in other countries, especially with regards to socialization with friends. This is possibly because in Argentina the intake of beer were among the top food sources of energy and grams intake [33]. Other clustering behaviors, including screen-time, occupational-time, and transportation-time while sitting were mixed among countries, with Peru and Colombia showing the highest prevalence. This suggests that public health policies and behavioral-change strategies, mainly in Peru and Colombia, should encourage more time spent on active activities, including those pertaining to the leisure and transportation physical activity (PA) domains. This may include more parks, fitness centers and other recreational facilities near to the participants’ homes, and encouragement to use motor-vehicles less for groceries and other shopping near to the individual home [34].

Concerning clustering of more than two unhealthy behaviors, subjects with lower educational and income-status background tend to present higher prevalence than their counterparts. This result may be explained by the inverse relationship between educational, income-status and age-group characteristics. Adolescents cannot be enrolled on any course higher then high-school degree and depend financially on their parents/caregivers and report more time on leisure screen-time and eating more unhealthy food sources (i.e., savory snacks, sweets and candies, and SSB) [31,35]. This result confirms that sedentary behaviors and energy-dense food items are inversely associated. Higher-educational status was associated with better diet quality and lower levels of sedentary behaviors. This corroborates with a pooled representative study, with data from six LA countries (Argentina, Brazil, Chile, Ecuador, Peru, and Surinam). Lower TV viewing as a proxy for sedentary behavior was found in subjects with greater educational background, mainly in Brazil [36]. Some studies use educational background as a proxy measurement for income status [28,37]. Individuals from high-income backgrounds are practicing more leisure-time PA, but at the same time performing more work-related sedentary activities. Thus, the time spent on PA may compensate for the seated time.

The prevalence of clustering behaviors in the current study is shown to be more common among diet-related behaviors, thus, corroborating previous study [11] and contradicting the idea that being active might be more important than diet for health [38]. As the individual gets older, it is harder to compensate for poor adherence to one healthy behavior with more favorable adherence to another healthy behavior [11]. Adults (compared to adolescents), despite meeting ≥5 recommendations for the dietary diversity score, reported a greater time spent sedentary-activities (mainly screen-time used for work-related activity), and/or drinking more alcoholic beverages. Sedentary behavior has been associated with all-cause mortality [39] and alcoholic beverages with cardiovascular diseases, certain types of cancers, and thus mortality [40]. From a clinical and public health perspective, promoting healthy eating, encouraging PA, reduce sitting time, and limiting SSB and alcohol intake should be considered in combination, as they play an important role in maintaining health and reducing risk of unhealthy weight gain, chronic non-communicable diseases and premature death [41].

In the logistic regression analyses, associations between ≥unhealthy behaviors and socio-demographic/weight status characteristics were mixed in terms of significance among LA countries. For example, all countries, with the exception of Brazil, did not remain significant for race/ethnicity background. Marital status only remained significant for Argentina, Colombia, and Venezuela. Weight status only showed significance for Argentina, Chile, and Peru. This may imply that some of the countries are moving in the right direction in public health policies and behavioral-change strategies incorporating important socio-demographic outcomes [10,11,36]. It is important to note that long-term consequences of preventive messages may have little effect on changing behavior [42], which may be explained by the fact that that people who engage in an EBRB are more focused on immediate rather than future consequences. There are possible explanations that can be drawn about the associations between personal characteristics and clustering of unhealthy behaviors. An individual who engages in an inadequate health behavior might experience decreased health, which may be linked to negative aspects of their personal characteristics, including socio-demographic factors, and reduce life-expectancy [10].

The strengths of this study include the use of a large, representative sample of eight LA countries, including adolescents and adults, and the examination of multiple health behaviors. However, it is not without limitations. First, the cross-sectional design of ELANS limits inferences related to temporality and causality. Second, the use of self-reported measurements of health behaviors might result in an under- or over-estimation of certain habits. Third, it was not possible to know how many people declined to participate in the survey, or dropped-out, as this information was not made available by the survey. Fourth, it was not possible to examine other health behaviors, including smoking and sleep, recognized risk factors for obesity and other NCDs [6,41], as these behaviors were not measured in the ELANS. Fifth, the occupational and transportation–sedentary time only assessed one behavior for each of these domains: reading-time and driving-time, respectively, thus assuming that these might be proxy measurements for these constructs. Finally, although the question used to assess SSB and alcohol intake has been used in prior publications [2,25] and has face validity, it was not formally validated.

## 5. Conclusions

EBRB, particularly excessive time spent on sedentary-activities and SSB intake, commonly co-occurred in a representative sample of LA adolescents and adults. While unhealthy behavior varied across LA countries, nearly half of sampled subjects in Argentina and Colombia presented at least two risk factor behaviors. Public health policies and behavioral-change strategies should target SB domains (screen-time, occupational, and transportation), diet intake, and SSB and alcoholic intake in combination. The overall high prevalence of these behaviors in the Latin America population underscores an important need for the promotion of healthy behaviors among these population groups.

## Figures and Tables

**Table 1:** Socio-demographics and lifestyle characteristics of the study sample: The Latin American Study of Nutrition and Health (ELANS) (*n* = 9.218).

	Total	Argentina	Brazil	Chile	Colombia	Costa Rica	Ecuador	Peru	Venezuela
Total *n*	1266	2000	879	1230	798	800	1113	1132	9218
Continuous variables (Mean ± SE)									
Age, years	35.82 ± 0.15	36.77 ± 0.39	36.51 ± 0.31	36.42 ± 0.48	36.93 ± 0.42	35.21 ± 0.49	34.25 ± 0.49	34.19 ± 0.41	34.99 ± 0.41
Body Mass Index, kg/m^2^	26.91 ± 0.06	27.09 ± 0.17	26.72 ± 0.13	28.08 ± 0.18	25.71 ± 0.14	27.65 ± 0.22	26.76 ± 0.19	26.65 ± 0.15	27.27 ± 0.17
Weight, kg	71.77 ± 0.17	73.56 ± 0.49	73.32 ± 0.38	74.96 ± 0.54	68.47 ± 0.43	73.11 ± 0.59	68.25 ± 0.51	67.46 ± 0.42	73.89 ± 0.51
Height, cm	163 ± 0.01	165 ± 0.00	165 ± 0.00	163 ± 0.00	163 ± 0.00	163 ± 0.00	160 ± 0.00	159 ± 0.00	164 ± 0.00
TV time, minutes/day	134.46 ± 1.18	136.06 ± 2.91	143.64 ± 2.99	114.95 ± 2.81	136.88 ± 3.39	178.87 ± 6.11	101.19 ± 2.43	136.76 ± 2.95	120.10 ± 2.28
Computer time, minutes/day	114.26 ± 2.31	96.72 ± 4.00	155.34 ± 7.23	105.48 ± 4.75	114.99 ± 5.78	153.29 ± 11.39	73.68 ± 3.63	105.65 ± 6.39	83.06 ± 3.30
Videogame time, minutes/day	90.26 ± 0.02	75.83 ± 6.61	114.56 ± 7.92	74.82 ± 7.63	87.79 ± 7.11	123.19 ± 9.46	70.00 ± 5.78	88.11 ± 7.64	72.31 ± 7.17
Reading time, minutes/day	55.29 ± 1.09	64.44 ± 3.00	71.81 ± 3.69	47.88 ± 2.99	56.35 ± 2.66	75.95 ± 5.23	38.13 ± 2.23	44.71 ± 1.74	39.51 ± 1.88
Socializing with friends, minutes/day	92.71 ± 1.15	107.04 ± 3.02	101.24 ± 2.77	73.09 ± 2.89	101.28 ± 3.46	133.17 ± 5.58	64.02 ± 2.53	74.90 ± 2.76	78.36 ± 2.36
Talking on the phone, minutes/day	45.48 ± 0.79	47.55 ± 1.88	48.07 ± 1.76	35.50 ± 2.26	53.46 ± 2.67	51.40 ± 3.19	30.46 ± 1.78	53.07 ± 2.25	34.66 ± 1.88
Driving time, minutes/day	87.25 ± 2.01	80.52 ± 4.48	77.58 ± 2.96	63.95 ± 3.87	102.78 ± 7.61	130.73 ± 10.53	79.61 ± 5.07	96.77 ± 10.71	95.53 ± 5.80
Screen time ^a^, minutes/day	192.65 ± 1.95	188.98 ± 4.25	224.97 ± 5.61	178.46 ± 4.84	193.13 ± 5.25	238.11 ± 8.58	140.17 ± 3.97	187.60 ± 4.92	160.13 ± 3.34
Leisure time ^b^, minutes/day	263.80 ± 2.42	272.78 ± 5.58	304.19 ± 6.73	229.18 ± 5.85	271.52 ± 6.69	331.73 ± 10.91	191.38 ± 5.00	244.47 ± 5.91	223.18 ± 4.37
Total Energy Intake, kcal/day	1992.93 ± 6.47	2181.07 ± 18.89	1835.55 ± 13.64	1732.72 ± 18.49	2130.43 ± 16.77	1886.07 ± 21.88	2212.55 ± 21.34	2111.04 ± 16.58	1917.83 ± 16.96
Categorical variables (*n* (%))									
Sex									
Female	4809 (52.17)	693 (54.74)	1058 (52.90)	454 (51.65)	627 (50.98)	404 (50.63)	403 (50.38)	590 (53.01)	580 (51.24)
Male	4409 (47.83)	573 (45.26)	942 (47.10)	425 (48.35)	603 (49.02)	394 (49.37)	397 (49.63)	523 (46.99)	552 (48.76)
Socio-economic status									
Low income	3856 (41.83)	616 (48.66)	261 (13.05)	411 (46.76)	779 (63.33)	262 (32.83)	114 (14.25)	533 (47.89)	880 (77.74)
Middle income	3946 (42.81)	585 (46.21)	1034 (51.70)	388 (44.14)	384 (31.22)	428 (53.68)	582 (72.75)	355 (31.90)	190 (16.78)
High income	1416 (15.36)	65 (5.13)	705 (35.25)	80 (9.10)	67 (5.45)	108 (13.53)	104 (13.00)	225 (20.22)	62 (5.48)
Educational background									
Don’t study	107 (1.16)	3 (0.24)	82 (4.10)	-	11 (0.89)	1 (0.13)	2 (0.25)	1 (0.09)	7 (0.62)
≤ high school	8233 (89.31)	1209 (95.50)	1750 (87.50)	780 (88.74)	1082 (87.97)	751 (94.11)	746 (93.25)	1003 (90.12)	912 (80.57)
College/University degree	878 (9.52)	54 (4.27)	168 (8.40)	99 (11.26)	137 (11.14)	46 (5.76)	52 (6.50)	109 (9.79)	213 (18.82)
Marital Status									
Single or living alone	4825 (52.34)	632 (49.92)	1071 (53.55)	473 (53.81)	668 (54.31)	430 (53.88)	386 (48.25)	526 (47.26)	639 (56.45)
Married or living partner	4393 (47.66)	634 (50.08)	929 (46.45)	406 (46.19)	562 (45.69)	368 (46.12)	414 (51.75)	587 (52.74)	493 (43.55)
Race/Ethnicity									
Caucasian	3216 (36.74)	856 (71.82)	797 (41.27)	279 (39.52)	290 (25.33)	394 (51.10)	39 (4.88)	96 (8.79)	462 (41.51)
Non-Caucasian	5537 (63.26)	337 (28.18)	1134 (58.73)	427 (60.48)	855 (74.67)	377 (48.90)	760 (95.12)	996 (91.21)	651 (58.49)
Weight Status									
Underweight	306 (3.32)	37 (2.92)	87 (4.35)	5 (0.57)	59 (4.80)	27 (3.38)	28 (3.50)	24 (2.18)	39 (3.45)
Normal weight	3420 (37.14)	493 (38.94)	749 (37.45)	271 (30.83)	548 (44.55)	267 (33.46)	288 (36.00)	414 (37.53)	390 (34.45)
Overweight	3167 (34.39)	399 (31.52)	664 (33.20)	332 (37.77)	419 (34.07)	260 (32.58)	287 (35.88)	422 (38.26)	384 (33.92)
Obese	2315 (25.14)	337 (26.62)	500 (25.00)	271 (30.83)	204 (16.59)	244 (30.58)	197 (24.63)	243 (22.03)	319 (28.18)
Screen-time									
Meeting	4690 (50.88)	670 (52.92)	1069 (53.45)	428 (48.69)	627 (50.98)	448 (56.14)	317 (39.63)	595 (53.46)	536 (47.35)
Not meeting	45.28 (49.12)	596 (47.08)	931 (46.55)	451 (51.31)	603 (49.02)	350 (43.86)	483 (60.38)	518 (46.54)	596 (52.65)
Leisure time									
Meeting	-	-	-	-	-	-	-	-	-
Not meeting	9218 (100.00)	1266 (100.00)	2000 (100.00)	879 (100.00)	1230 (100.00)	798 (100.00)	800 (100.00)	1113 (100.00)	1132 (100.00)
Occupation									
Meeting	3032 (32.89)	434 (34.28)	600 (30.00)	250 (28.44)	457 (37.15)	282 (35.34)	219 (27.38)	473 (42.50)	317 (28.00)
Not meeting	6186 (67.11)	832 (65.72)	1400 (70.00)	629 (71.56)	773 (62.85)	516 (64.66)	581 (72.63)	640 (57.50)	815 (72.00)

Note: All variables presented significant differences between countries, with the exception of that for sex. Significant differences were found using appropriate tests. ^a^ Screen-time is the sum of time spent on TV and computer. ^b^ Leisure-time is the sum of time spent on TV, computer, talking on the phone, and socializing (being) with friend. Screen, leisure and occupation time on sedentary behavior: meeting recommendations were established based on the median of these variables. MVPA: Moderate-to-vigorous Physical Activity.

**Table 2:** Patterns of sedentary behaviors and dietary components among adolescents and adults. The Latin American Health and Nutrition Study (ELANS) (*n* = 9218).

	Argentina	Brazil	Chile	Colombia	Costa Rica	Ecuador	Peru	Venezuela	ELANS
Total *n*	1266	2000	879	1230	798	800	1113	1132	9218
Number of unhealthy behaviors									
Clustering (≥2 unhealthy behaviors)	48.74%	39.50%	35.49%	49.27%	45.36%	33.88%	40.97%	34.28%	41.25%
Prevalence of unhealthy behaviors pairs									
Screen time + occupation time	19.43%	18.75%	15.36%	20.81%	21.43%	13.38%	25.52%	15.19%	18.94%
Screen time + transportation time	9.08%	11.75%	7.74%	6.10%	10.90%	9.38%	4.49%	8.75%	8.72%
Screen time + poor diet	26.46%	29.65%	31.06%	29.84%	35.46%	29.88%	38.54%	22.44%	30.08%
Screen time + SSB	35.70%	31.95%	22.18%	38.05%	35.84%	19.13%	28.21%	26.86%	30.49%
Screen time + alcohol	36.10%	28.10%	28.10%	34.07%	26.32%	24.25%	32.97%	28.18%	30.10%
Socialization with friends + screen time	33.33%	30.80%	22.18%	30.41%	30.45%	18.88%	24.98%	25.53%	27.95%
Socialization with friends + occupation time	20.85%	17.25%	12.29%	21.46%	19.17%	11.13%	18.24%	12.63%	17.02%
Socialization with friends + transportation time	9.16%	11.25%	6.71%	6.10%	10.28%	6.50%	3.41%	7.95%	8.00%
Occupation time + Transportation time	5.92%	7.00%	4.66%	3.90%	6.52%	5.50%	3.68%	6.27%	5.55%
Socialization with friends + poor diet	28.20%	26.20%	20.93%	29.59%	32.23%	25.13%	26.68%	21.38%	26.33%
Socialization with friends + SSB	37.05%	29.80%	16.27%	37.32%	32.71%	17.38%	19.68%	26.15%	28.01%
Socialization with friends + alcohol	39.18%	27.40%	22.98%	32.76%	21.93%	21.88%	23.27%	26.41%	27.74%
Occupation time + poor diet	18.48%	18.35%	17.75%	24.55%	24.31%	20.63%	30.73%	12.72%	20.66%
Occupation time + SSB	21.17%	17.90%	12.63%	27.15%	22.56%	12.83%	21.56%	16.87%	19.34%
Occupation time + alcohol	21.88%	13.65%	16.72%	22.93%	14.41%	15.25%	23.90%	14.49%	17.86%
Transportation time + poor diet	8.53%	11.70%	9.44%	6.91%	11.90%	13.00%	5.03%	8.30%	9.32%
Transportation time + SSB	10.43%	11.25%	8.08%	8.54%	12.28%	7.38%	3.95%	10.51%	9.25%
Transportation time + alcohol	13.19%	10.90%	11.04%	7.97%	9.65%	10.38%	5.12%	11.31%	10.03%
Poor diet + SSB	32.94%	33.30%	28.44%	45.20%	40.60%	35.00%	35.76%	26.24%	34.58%
Poor diet + alcohol	34.52%	26.95%	37.54%	36.75%	28.95%	39.88%	42.86%	26.33%	33.45%
SSB + Alcohol	45.50%	30.15%	29.12%	48.29%	28.45%	28.75%	31.81%	33.22%	34.89%

Poor diet: not meeting five of recommendations of the Dietary Diversity Score.

**Table 3:** Estimating association ^1^ with unhealthy behavior clustering across socio-demographic categories. The Latin American Health and Nutrition Study (ELANS) (*n* = 9218).

	ELANS	Argentina	Brazil	Chile	Colombia
OR (95%CI)					
Age (years)	0.01 (0.01, 0.02) ***	0.011 (0.00; 0.02) **	0.01 (0.01; 0.02) ***	−0.14 (−0.52, 0.24)	0.02 (0.01, 0.03) ***
Sex					
Female	0.59 (0.51, 0.68) ***	0.55 (0.33, 0.78) ***	0.59 (0.41, 0.77) ***	0.56 (0.28, 0.84) ***	0.59 (0.36, 0.82) ***
Male	Ref	Ref	Ref	Ref	Ref
Educational Background					
≤ High school	0.68 (0.54, 0.82) ***	0.80 (0.21, 1.39) **	1.19 (0.86, 1.53) ***	0.67 (0.21, 1.13) **	0.66 (0.28, 1.04) ***
College/University	Ref	Ref	Ref	Ref	Ref
Marital Status					
Single or living alone	0.27 (0.35, 0.18) ***	0.23 (0.45, −0.00)	0.08 (0.27, −0.09)	0.27 (0.58, −0.03)	0.44 (0.67, 0.20) **
Married or living with partner	Ref				
Race/Ethnicity					
White	0.01 (−0.08, 0.09)	0.34 (0.09, 0.59) **	0.04 (−0.14, 0.23)	0.02 (−0.29, 0.34)	−0.05 (−0.30, 0.21)
Non-White	Ref	Ref	Ref	Ref	Ref
SES					
Low	0.66 (0.53, 0.78) ***	0.82 (0.30, 1.37) **	1.04 (0.73, 1.36) ***	0.97 (0.49, 1.46) ***	0.62 (0.11, 1.14) *
Middle	0.40 (0.28, 0.53) ***	0.54 (0.02, 1.09) *	0.50 (0.31, 0.69) ***	0.57 (0.09, 1.06) **	0.10 (−0.42, 0.64)
High	Ref	Ref	Ref	Ref	Ref
Weight Status					
Underweight	0.15 (0.39, −0.10)	0.03 (0.72, −0.66)	0.07 (0.53, −0.40)	---	0.26 (0.84, −0.32)
Normal Weight	0.18 (0.30, 0.08) **	0.23 (0.51, −0.04)	0.07 (0.16, −0.30)	0.52 (0.88, 0.15) **	0.25 (0.57, −0.07)
Overweight	0.08 (0.19, −0.03)	0.17 (0.46, −0.12)	0.06 (0.29, −0.18)	0.51 (0.88, 0.15) **	0.02 (0.36, −0.32)
Obese	Ref	Ref	Ref	Ref	Ref
	**Costa Rica**	**Ecuador**	**Peru**	**Venezuela**	
**OR (95%CI)**					
Age (years)	0.02 (0.01, 0.03) **	0.02 (0.01, 0.03) ***	0.01 (0.01, 0.02) **	0.01 (0.00, 0.02) *	
Sex					
Female	0.74 (0.46, 1.03) ***	0.88 (0.58, 1.18) ***	0.66 (0.42, 0.90) ***	0.42 (0.18, 0.67) **	
Male	Ref	Ref	Ref	Ref	
Educational Background					
≤High school	0.64 (0.03, 1.26) *	0.97 (0.39, 1.54) **	0.42 (0.02, 0.82) *	0.66 (0.36, 0.97) ***	
College/University	Ref	Ref	Ref	Ref	
Marital Status					
Single or living alone	0.42 (0.70, 0.13) **	−0.47 (−0.77, −0.18) **	−0.42 (−0.69, −0.21) **	−0.05 (−0.31, 0.19)	
Married or living with partner	Ref	Ref	Ref	Ref	
Race/Ethnicity					
White	0.14 (−0.14, 0.43)	0.03 (−0.64, 0.74)	0.27 (−0.17, 0.71)	0.15 (−0.11, 0.39)	
Non-White	Ref	Ref	Ref	Ref	
SES					
Low	0.96 (0.49, 1.42) ***	1.26 (0.68, 1.85) ***	0.95 (0.63, 1.26) ***	0.57 (0.04, 1.08) *	
Middle	0.20 (−0.23, 0.61)	0.75 (0.33, 1.17) **	0.73 (0.39, 1.07) ***	0.50 (−0.08, 1.08)	
High	Ref	Ref	Ref	Ref	
Weight Status					
Underweight	0.19 (0.61, −1.03)	0.05 (0.88, −0.84)	0.77 (1.63, −0.08)	0.39 (0.32, −1.16)	
Normal Weight	0.07 (0.42, −0.28)	0.39 (0.78, 0.01) *	0.49 (0.81, 0.16) **	0.19 (0.12, −0.51)	
Overweight	0.07 (0.29, −0.42)	0.05 (−0.35, 0.44)	−0.09 (−0.42, 0.24)	0.08 (0.23, −0.39)	
Obesity	Ref	Ref	Ref	Ref	

Note: Ref: reference, SES: Socio-economic status, OR: Odds Ratio. Bold values are significant: * *p* < 0.05, ** *p* < 0.01, and *** *p* < 0.001. Results are from logistic regression models including all variables shown above. ^1^ Marginal standardization was used to calculate standardized proportions, which can be interpreted as the proportion of participants who have at least 2 unhealthy behaviors if standardized to the characteristics of the entire sample.

## Data Availability

No new data were created or analyzed in this study. Data sharing is not applicable to this article.

## References

[1] Blüher M. (2019). Obesity: Global epidemiology and pathogenesis. Nat. Rev. Endocrinol..

[2] Kovalskys I., Fisberg M., Gomez G., Pareja R.G., Yepez Garcia M.C., Cortes Sanabria L.Y., Herrera-Cuenca M., Rigotti A., Guajardo V., Zalcman Zimberg I. (2018). Energy intake and food sources of eight Latin American countries: Results from the Latin American Study of Nutrition and Health (ELANS). Public Health Nutr..

[3] Finck Barboza C., Monteiro S.M., Barradas S.C., Sarmiento O.L., Rios P., Ramirez A., Mahecha M.P., Pratt M. (2013). Physical activity, nutrition and behavior change in Latin America: A systematic review. Glob. Health Promot..

[4] Malta D.C., Duncan B.B., Schmidt M.I., Teixeira R., Ribeiro A.L.P., Felisbino-Mendes M.S., Machado Í.E., Velasquez-Melendez G., Brant L.C.C., Silva D.A.S. (2020). Trends in mortality due to non-communicable diseases in the Brazilian adult population: National and subnational estimates and projections for 2030. Popul. Health Metr..

[5] Perez-Ferrer C., Auchincloss A.H., de Menezes M.C., Kroker-Lobos M.F., Cardoso L.O., Barrientos-Gutierrez T. (2019). The food environment in Latin America: A systematic review with a focus on environments relevant to obesity and related chronic diseases. Public Health Nutr..

[6] Dinkel D., Lu K., John J., Snyder K., Jacobson L.T. (2021). A Cross-Sectional Examination of Physical Activity, Sedentary Time, and Sleep Between Adults with and without Children in the Home Using National Health and Nutrition Examination Survey. J. Phys. Act. Health.

[7] Du Y., Liu B., Sun Y., Snetselaar L.G., Wallace R.B., Bao W. (2019). Trends in Adherence to the Physical Activity Guidelines for Americans for Aerobic Activity and Time Spent on Sedentary Behavior Among US Adults, 2007 to 2016. JAMA Netw. Open.

[8] Leech R.M., McNaughton S.A., Timperio A. (2014). The clustering of diet, physical activity and sedentary behavior in children and adolescents: A review. Int. J. Behav. Nutr. Phys. Act..

[9] Mayne S.L., Virudachalam S., Fiks A.G. (2020). Clustering of unhealthy behaviors in a nationally representative sample of U.S. children and adolescents. Prev. Med..

[10] Dieteren C.M., Brouwer W.B.F., van Exel J. (2020). How do combinations of unhealthy behaviors relate to attitudinal factors and subjective health among the adult population in the Netherlands?. BMC Public Health.

[11] Del Pozo Cruz B., McGregor D.E., Del Pozo Cruz J., Buman M.P., Palarea-Albaladejo J., Alfonso-Rosa R.M., Chastin S.F.M. (2020). Integrating Sleep, Physical Activity, and Diet Quality to Estimate All-Cause Mortality Risk: A Combined Compositional Clustering and Survival Analysis of the National Health and Nutrition Examination Survey 2005–2006 Cycle. Am. J. Epidemiol..

[12] Ding D., Rogers K., van der Ploeg H., Stamatakis E., Bauman A.E. (2015). Traditional and Emerging Lifestyle Risk Behaviors and All-Cause Mortality in Middle-Aged and Older Adults: Evidence from a Large Population-Based Australian Cohort. PLoS Med..

[13] Felez-Nobrega M., Raine L.B., Haro J.M., Wijndaele K., Koyanagi A. (2020). Temporal trends in leisure-time sedentary behavior among adolescents aged 12–15 years from 26 countries in Asia, Africa, and the Americas. Int. J. Behav. Nutr. Phys. Act..

[14] Huybrechts I., Lioret S., Mouratidou T., Gunter M.J., Manios Y., Kersting M., Gottrand F., Kafatos A., de Henauw S., Cuenca-Garcia M. (2017). Using reduced rank regression methods to identify dietary patterns associated with obesity: A cross-country study among European and Australian adolescents. Br. J. Nutr..

[15] Batis C., Mazariegos M., Martorell R., Gil A., Rivera J.A. (2020). Malnutrition in all its forms by wealth, education and ethnicity in Latin America: Who are more affected?. Public Health Nutr..

[16] Salmon C.T., Nichols J.S. (1983). The Next-Birthday Method of Respondent Selection. Public Opin. Q..

[17] (2008). Encyclopedia of Survey Research Methods.

[18] Fisberg M., Kovalskys I., Gomez G., Rigotti A., Cortes L.Y., Herrera-Cuenca M., Yepez M.C., Pareja R.G., Guajardo V., Zimberg I.Z. (2016). Latin American Study of Nutrition and Health (ELANS): Rationale and study design. BMC Public Health.

[19] WHO (2021). Adolescence Health: Overview.

[20] De Onis M., Onyango A.W., Borghi E., Siyam A., Nishida C., Siekmann J. (2007). Development of a WHO growth reference for school-aged children and adolescents. Bull. World Health Organ..

[21] WHO (2021). Body Mass Index—BMI.

[22] Loyen A., Chau J.Y., Jelsma J.G.M., van Nassau F., van der Ploeg H.P. (2019). Prevalence and correlates of domain-specific sedentary time of adults in the Netherlands: Findings from the 2006 Dutch time use survey. BMC Public Health.

[23] Moshfegh A.J., Rhodes D.G., Baer D.J., Murayi T., Clemens J.C., Rumpler W.V., Paul D.R., Sebastian R.S., Kuczynski K.J., Ingwersen L.A. (2008). The US Department of Agriculture Automated Multiple-Pass Method reduces bias in the collection of energy intakes. Am. J. Clin. Nutr..

[24] Harnack L., Gellman M.D., Turner J.R. (2013). Nutrition Data System for Research (NDSR). Encyclopedia of Behavioral Medicine.

[25] Kovalskys I., Fisberg M., Gomez G., Rigotti A., Cortes L.Y., Yepez M.C., Pareja R.G., Herrera-Cuenca M., Zimberg I.Z., Tucker K.L. (2015). Standardization of the Food Composition Database Used in the Latin American Nutrition and Health Study (ELANS). Nutrients.

[26] Gomez G., Fisberg R.M., Nogueira Previdelli A., Hermes Sales C., Kovalskys I., Fisberg M., Herrera-Cuenca M., Cortes Sanabria L.Y., Garcia M.C.Y., Pareja Torres R.G. (2019). Diet Quality and Diet Diversity in Eight Latin American Countries: Results from the Latin American Study of Nutrition and Health (ELANS). Nutrients.

[27] Kennedy G., Ballard T., Dop M.C. (2013). Guidelines for Measuring Household and Individual Dietary Diversity.

[28] Leme A.C., Muszynski D., Mirotta J.A., Carrol N., Hogan J., Jewell K., Yu J., Fisberg R., Duncan A., Ma D. (2021). Diet quality of Canadian preschool children: Associations with socio-demographic characteristics. Can. Diet. J. Pract. Res..

[29] Food and Agriculture of the United Nations (2016). Minimum Dietary Diversity for Women. A Guide for Measurement.

[30] Leme A.C., Baranowski T., Thompson D., Philippi S., O’Neil C.E., Fulgoni V.L., Nicklas T.A. (2020). Food Sources of Shortfall Nutrients Among US Adolescents: National Health and Nutrition Examination Survey (NHANES) 2011–2014. Fam. Community Health.

[31] Leme A.C., Baranowski T., Thompson D., Philippi S., O’Neil C., Fulgoni V., Nicklas T. (2019). Top food sources of percentage of energy, nutrients to limit and total gram amount consumed among US adolescents: National Health and Nutrition Examination Survey 2011–2014. Public Health Nutr..

[32] Fausnacht A.G., Myers E.A., Hess E.L., Davy B.M., Hedrick V.E. (2020). Update of the BEVQ-15, a beverage intake questionnaire for habitual beverage intake for adults: Determining comparative validity and reproducibility. J. Hum. Nutr. Diet..

[33] Fisberg R., Leme A.C., Nogueira A.P., Veroneze A.M., Arroyo A.M., Sales C.H., Gomez G.S., Kovalskys I., Cortes Sanabria L.Y., Herrera-Cuenca M. (2021). Contribution of food groups to energy, grams, and nutrients-to-limit: The Latin American Study of Nutrition and Health/Estudio Latino Americano de Nutricíon y Salud (ELANS). Public Health Nutr..

[34] Habinger J.G., Chavez J.L., Matsudo S.M., Kovalskys I., Gomez G., Rigotti A., Sanabria L.Y.C., Garcia M.C.Y., Pareja R.G., Herrera-Cuenca M. (2020). Active Transportation and Obesity Indicators in Adults from Latin America: ELANS Multi-Country Study. Int J. Environ. Res. Public Health.

[35] Perera M.J., Chirinos D.A., Brintz C.E., Schneiderman N., Daviglus M., Talavera G.A., Perreira K.M., Giacinto R.A.E., Qi Q., Llabre M.M. (2020). Body Mass of U.S. Hispanics/Latinos From the Hispanic Community Health Study/Study of Latinos (HCHS/SOL): How Do Diet Quality and Sedentary Time Relate?. Hisp. Health Care Int..

[36] Werneck A.O., Baldew S.S., Miranda J.J., Diaz Arnesto O., Stubbs B., Silva D.R., South American Physical Activity and Sedentary Behavior Network (SAPASEN) (2019). Physical activity and sedentary behavior patterns and sociodemographic correlates in 116,982 adults from six South American countries: The South American physical activity and sedentary behavior network (SAPASEN). Int. J. Behav. Nutr. Phys. Act..

[37] Mello A.V., Pereira J.L., Leme A.C.B., Goldbaum M., Cesar C.L.G., Fisberg R.M. (2020). Social determinants, lifestyle and diet quality: A population-based study from the 2015 Health Survey of Sao Paulo, Brazil. Public Health Nutr..

[38] Katzmarzyk P.T., Barreira T.V., Broyles S.T., Champagne C.M., Chaput J.-P., Fogelholm M., Hu G., Johnson W.D., Kuriyan R., Kurpad A. (2015). Relationship between lifestyle behaviors and obesity in children ages 9–11: Results from a 12-country study. Obesity.

[39] Park J.H., Moon J.H., Kim H.J., Kong M.H., Oh Y.H. (2020). Sedentary Lifestyle: Overview of Updated Evidence of Potential Health Risks. Korean J. Fam Med..

[40] Zhang X., Liu Y., Li S., Lichtenstein A.H., Chen S., Na M., Veldheer S., Xing A., Wang Y., Wu S. (2021). Alcohol consumption and risk of cardiovascular disease, cancer and mortality: A prospective cohort study. Nutr. J..

[41] Zheng H., Echave P. (2021). Are Recent Cohorts Getting Worse? Trends in U.S. Adult Physiological Status, Mental Health, and Health Behaviors across a Century of Birth Cohorts. Am. J. Epidemiol..

[42] Leme A.C., Philippi S.T. (2015). The “Healthy Habits, Healthy Girls” randomized controlled trial for girls: Study design, protocol, and baseline results. Cad. Saude Publica.

